# Supplementary material for: Stabilization of GTSE1 by cyclin D1-CDK4/6-mediated phosphorylation promotes cell proliferation: relevance in cancer prognosis
Source: bioRxiv. 2025 Jan 23:2024.06.26.600797. Originally published 2024 Jun 27. Preprint. [Version 2] doi: 10.1101/2024.06.26.600797 (PMC11230433; doi:10.1101/2024.06.26.600797)
Supplement: Supplement 1 [file NIHPP2024.06.26.600797v2-supplement-1.pdf]

bioRxiv preprint doi: https://doi.org/10.1101/2024.06.26.600797; this version posted January 23, 2025. The copyright holder for this preprint (which was not certified by peer review) is the author/funder, who has granted bioRxiv a license to display the preprint in perpetuity. It is made available under aCC-BY 4.0 International license.

Figure Supplement Legends

Figure Supplement 1

A) Schematic of the bioinformatics workflow implemented to identify potential substrates of the D-type cyclins. The process integrates proteomic data from AMBRA1 knockout (KO) U2OS cells versus parental cells[11], protein annotations of the CDK phosphorylation consensus site [S/T*]PX[K/R] from PhosphoSitePlus[24], and cancer-related databases.

B) Kaplan-Meier curves representing the overall survival analysis based on the 50% upper versus lower expression levels of GTSE1. Survival analysis was conducted across the indicated cancer cohorts. The hazard ratio (HR) was calculated to estimate the relative risk, and significance was assessed using the log-rank test with a threshold of $p<0.05$. Curves were generated using the GEPIA2 platform [42].

Figure Supplement 2

A) Phylogenetic Conservation of GTSE1 Phosphorylation Sites Across Species. Illustration of the evolutionary conservation of phosphorylation sites within the GTSE1 protein.

B) The sequence alignment highlights identical (red) and similar residues (orange) across a range of species, indicating the conserved nature of these phosphorylation sites in GTSE1 orthologs.

Figure Supplement 3

A) HEK293T cells transfected with the indicated single Ser-to-Ala mutants of HA-tagged GTSE1, with and without Cyclin D1-CDK4 co-expression, followed by analysis of their migration pattern on phos-tag™ gels.

B) Immunoblot analysis displaying cell cycle synchronization effects following 72 hrs of serum starvation in parental T98G cells and AMBRA1 KO T98G cells. Cells were collected at various time points post-serum re-supplementation as indicated, followed by immunoblotting with the indicated antibodies. Cyclin A2 and Cyclin B1 serve as S phase and G2-M markers, respectively. pH3 at Ser10 serves as a mitotic marker. GAPDH serves a loading control.

C) Heatmap illustrating differential abundance of GTSE1 phosphorylation in cancer using CPTAC data[44]. Color intensity reflects the log2-transformed Z-scores of identified GTSE1 phosphopeptides, indicating relative phosphorylation levels across various tumor cohorts compared to adjacent normal tissues.

D) HEK293T cells were subjected to transfection with the indicated variants of HA-tagged GTSE1, with and without FFSS-Cyclin D1-CDK4 co-expression. Where indicated, cells were treated with Palbociclib for 4 hrs before harvest. Finally, samples were subjected to western blot analysis with the indicated antibodies.

E) HCT mAID FLAG-AMBRA KI cells underwent pull-down using an anti-FLAG antibody versus an IgG sham pull-down. Where indicated, cells were treated with MLN4924 for 3 hours prior to harvesting. Western blot analysis was performed using the specified antibodies to compare the pull-down fraction with the whole cell extract (WCE). Vinculin serves as WCE loading control.

Figure Supplement 4

A) Bubble plot derived from CPTAC data depicting the correlation GTSE1 protein abundance with the levels of proteins involved in cell proliferation or cell migration across various cancer types. Data analysis was performed as detailed in Figure 4A.

bioRxiv preprint doi: https://doi.org/10.1101/2024.06.26.600797; this version posted January 23, 2025. The copyright holder for this preprint (which was not certified by peer review) is the author/funder, who has granted bioRxiv a license to display the preprint in perpetuity. It is made available under aCC-BY 4.0 International license.

B) Growth curves comparing the proliferation of AMBRA1 KO clones with that of parental U2OS cells, with graphs displaying mean cell counts at each time point, n=3 biological replicates per clone. Statistical significance was determined using unpaired T-tests with p-values<0.05. Error bars represent SEM.

C) Gating strategy for FACS analysis shown in Figure 4C. The gating strategy was implemented to identify live, single cells positive for GFP. The fourth panel (proliferation analysis) is the same as the first panel in Figure 4C (GTSE1), which is used here to show the gating strategy.

D-E) Independent biological replicates of cell proliferation analysis using CellTrace™ as shown in Figure 4C. Generation distribution in D: WT GTSE1 (Undivided: 2.6%, Gen 1: 8.5%, Gen 2: 44.0%, Gen 3: 39.7%, Gen 4: 5.4%), Tetra SA GTSE1 (Undivided: 0.1%, Gen 1: 5.5%, Gen 2: 44.5%, Gen 3: 50.0%, Gen 4: 0%), and Tetra SD GTSE1 (Undivided: 1.0%, Gen 1: 4.5%, Gen 2: 40.0%, Gen 3: 34.4%, Gen 4: 20.0%).Generation distribution in E: WT GTSE1 (Undivided: 16.8%, Gen 1: 52.9%, Gen 2: 27.9%, Gen 3: 2.5%, Gen 4: 0.3%), Tetra SA GTSE1 (Undivided: 22.0%, Gen 1: 49.7%, Gen 2: 24.8%, Gen 3: 3.3%, Gen 4: 0%), and Tetra SD GTSE1 (Undivided: 10.0%, Gen 1: 38.0%, Gen 2: 40.0%, Gen 3: 10.1%, Gen 4: 1.5%).

F) Representative cell cycle analysis of U2OS cells expressing wild-type (WT), Tetra SA, and Tetra SD constructs. Cells were pulsed with EdU and stained with propidium iodide (PI) for DNA content assessment. Cell cycle distribution was quantified by FACS using the gating strategy described in panel C. Graph on the right shows the percentage of cells in each phase of the cell cycle: AMBRA Parental (G1: 42.9%, S: 46.2%, G2/M: 10.8%), AMBRA KO (G1: 35.0%, S: 51.7%, G2/M: 13.0%), WT GTSE1 (G1: 45.0%, S: 43.8%, G2/M: 11.2%), Tetra SA GTSE1 (G1: 45.4%, S: 42.2%, G2/M: 11.8%), and Tetra SD GTSE1 (G1: 38.6%, S: 46.6%, G2/M: 14.0%).

bioRxiv preprint doi: https://doi.org/10.1101/2024.06.26.600797; this version posted January 23, 2025. The copyright holder for this preprint (which was not certified by peer review) is the author/funder, who has granted bioRxiv a license to display the preprint in perpetuity. It is made available under aCC-BY 4.0 International license.

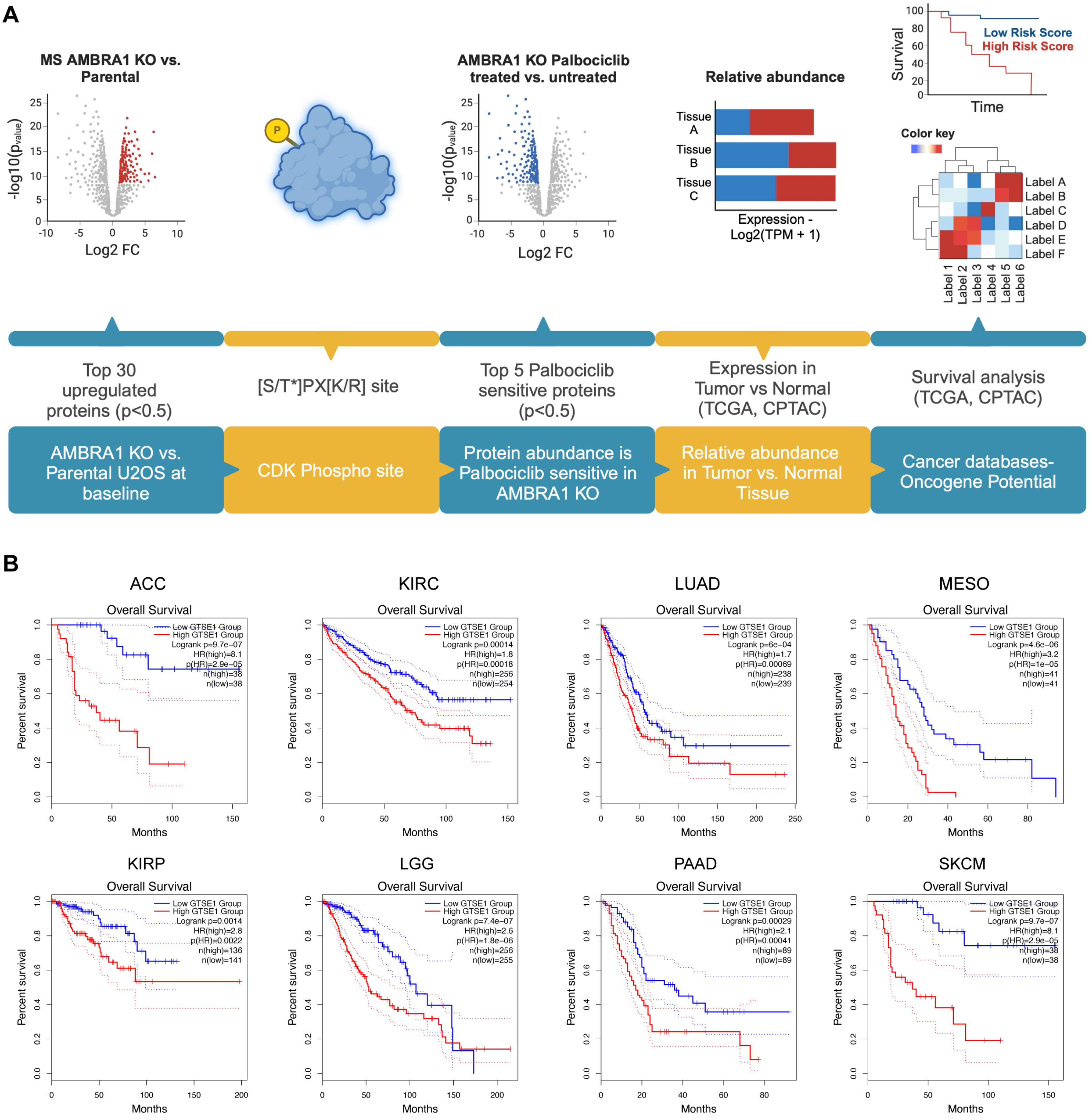

Figure Supplement 1 - García-Vázquez et al.

bioRxiv preprint doi: https://doi.org/10.1101/2024.06.26.600797; this version posted January 23, 2025. The copyright holder for this preprint (which was not certified by peer review) is the author/funder, who has granted bioRxiv a license to display the preprint in perpetuity. It is made available under aCC-BY 4.0 International license.

A

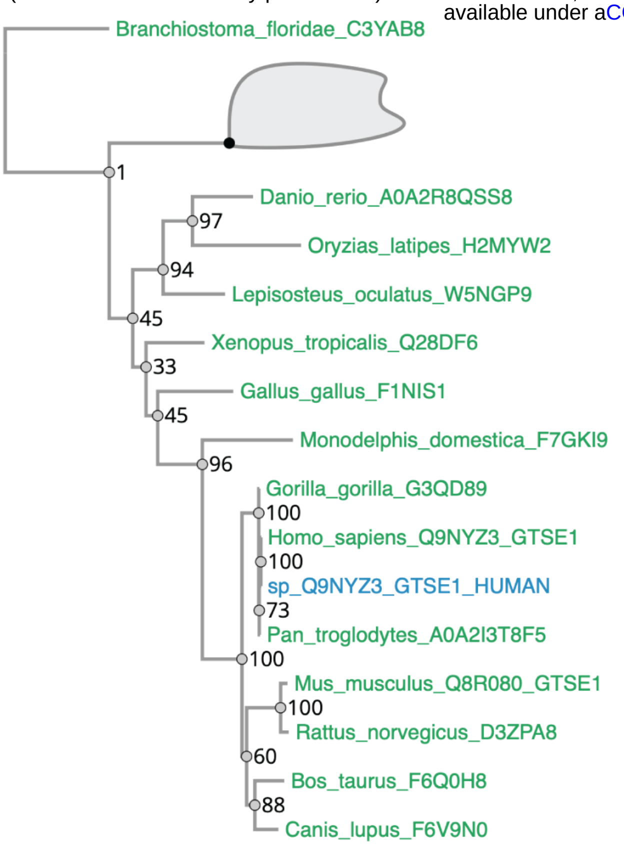

B

Site	Species	Sequence
S86	Homo sapiens	PPLPTSESPFAWSPL
	Rattus norvegicus	PLAPGSGSPCTWSPL
	Mus musculus	PLAPGSGSPCTLSPL
	Gallus gallus	KRVPISDEKLTWSPL
	Branchiostoma floridae	-----EEEKFVPLEL
S91	Homo sapiens	SESPFAWSPLAGEKF
	Rattus norvegicus	SGSPCTWSPLTGEKF
	Mus musculus	SGSPCTLSPLTGEKF
	Gallus gallus	SDEKLTWSPLTGDKF
	Branchiostoma floridae	EEEKFVPLELNGDQY
S157	Homo sapiens	KEKEMKKSPTSLKRE
	Rattus norvegicus	EQKRDKSPLSLKRE
	Mus musculus	KEQKRDRSPMSLKRE
	Gallus gallus	HH-IEKSPRAVKRE
	Branchiostoma floridae	KAPQNSPRNSPRRM
S171	Homo sapiens	ETYYLSDSP--LLGPPV
	Rattus norvegicus	ETFCLPSS---RAQPLL
	Mus musculus	ETFCLPSS---RV-QPPM
	Gallus gallus	ETFCVGDSPacMLLPSFQ
	Branchiostoma floridae	MTYTLDT[5]ASssTTTADVQ
S187	Homo sapiens	EPRLLASSPAL--PSSG
	Rattus norvegicus	EPQLLA-SPGLPNS-
	Mus musculus	EPQLLA-SPGLLSS-
	Gallus gallus	KESDKL----LPGDK
	Branchiostoma floridae	DSTQRQeNNEVKEGQ
S262	Homo sapiens	PKKEIPASPSRTKIP
	Rattus norvegicus	LKKEVPASLQQTKPL
	Mus musculus	LKKEVPASIQRTKLV
	Gallus gallus	AKPGRQTNISTKRDS
	Branchiostoma floridae	IKPGI-------RK[16]A
S345	Homo sapiens	GASSACTSPAVGKAK
	Rattus norvegicus	LVSGVGRSPTAGKAK
	Mus musculus	LESGVYRSSVAGKAK
	Gallus gallus	SVSSNLNSSL[4]IGKNG
	Branchiostoma floridae	TNPSQGSLGR[3]NGSID
S454	Homo sapiens	RSIRRRDSCLNSKTK
	Rattus norvegicus	VSIKRQDSSLGCKTE
	Mus musculus	VSIKRRDSYLSCKTE
	Gallus gallus	GSTTKGSLCPKPRAR
	Branchiostoma floridae	AASANTSFSKKSLLP
S480	Homo sapiens	PKFSIGDSPDSSTPK
	Rattus norvegicus	PQFSVGESPDRVTPK
	Mus musculus	PQFSVGESPGGVTPK
	Gallus gallus	PVKAGDITPNKSATK
	Branchiostoma floridae	PMRATLPMSQISTPK
S555	Homo sapiens	TMPRAVGSPLCVPAR
	Rattus norvegicus	SMPRALVSPLCVPAR
	Mus musculus	SMPRALVSPLCVPAR
	Gallus gallus	TTPRTVFSPRIESVR
	Branchiostoma floridae	SSRRRSGIPTPRSMQ
S594	Homo sapiens	DSRLVDVSPDRGSPP
	Rattus norvegicus	GQSQGLSSDESSSPP
	Mus musculus	GQAQGLSSDESSSPP
	Gallus gallus	------SSEDDPSPP
	Branchiostoma floridae	SCLPTLAANDSPSEF
S599	Homo sapiens	DVSPDRGSPPSRVPQ
	Rattus norvegicus	LSSDESSSPPSSVPQ
	Mus musculus	LSSDESSSPPSSVPQ
	Gallus gallus	*-SSEDDPSPPPVLPL
	Branchiostoma floridae	LAANDSPSEFPATHL
S611	Homo sapiens	VPQALNFSPEESDST
	Rattus norvegicus	VPQALNFSPEKSDFP
	Mus musculus	VPQALNFSPEKSASP
	Gallus gallus	LPLMLDFSPEKAVI-
	Branchiostoma floridae	THLR-----------
S707	Homo sapiens	NK-NVAKPSPV-VGQLI
	Rattus norvegicus	GRSDVGKPPKAELGQLI
	Mus musculus	GRNDVGKPAKAELGQLI
	Gallus gallus	NKVIPLKPVFSEQIKLI
	Branchiostoma floridae	-----------------
S718	Homo sapiens	GQLIDLSSPLIQLSP
	Rattus norvegicus	GQLIDLGSPLIQLSP
	Mus musculus	GQLIDLGSPLIQLSP
	Gallus gallus	IKLIDLSSPLITLSP
	Branchiostoma floridae	-------KPLIDFGE
S724	Homo sapiens	SSPLIQLSPEADKEN
	Rattus norvegicus	GSPLIQLSPEADKEN
	Mus musculus	GSPLIQLSPEADKEN
	Gallus gallus	SSPLITLSPDINKEN
	Branchiostoma floridae	-KPLIDFGEEDMKTN
S734	Homo sapiens	ADKENVDSPLLKF
	Rattus norvegicus	ADKENVDSPLLKF
	Mus musculus	ADKENVDSPLLKF
	Gallus gallus	INKENLDSPLLKF
	Branchiostoma floridae	DMKTN--SPAIAN

Figure Supplement 2 - García-Vázquez et al.

bioRxiv preprint doi: https://doi.org/10.1101/2024.06.26.600797; this version posted January 23, 2025. The copyright holder for this preprint (which was not certified by peer review) is the author/funder, who has granted bioRxiv a license to display the preprint in perpetuity. It is made available under aCC-BY 4.0 International license.

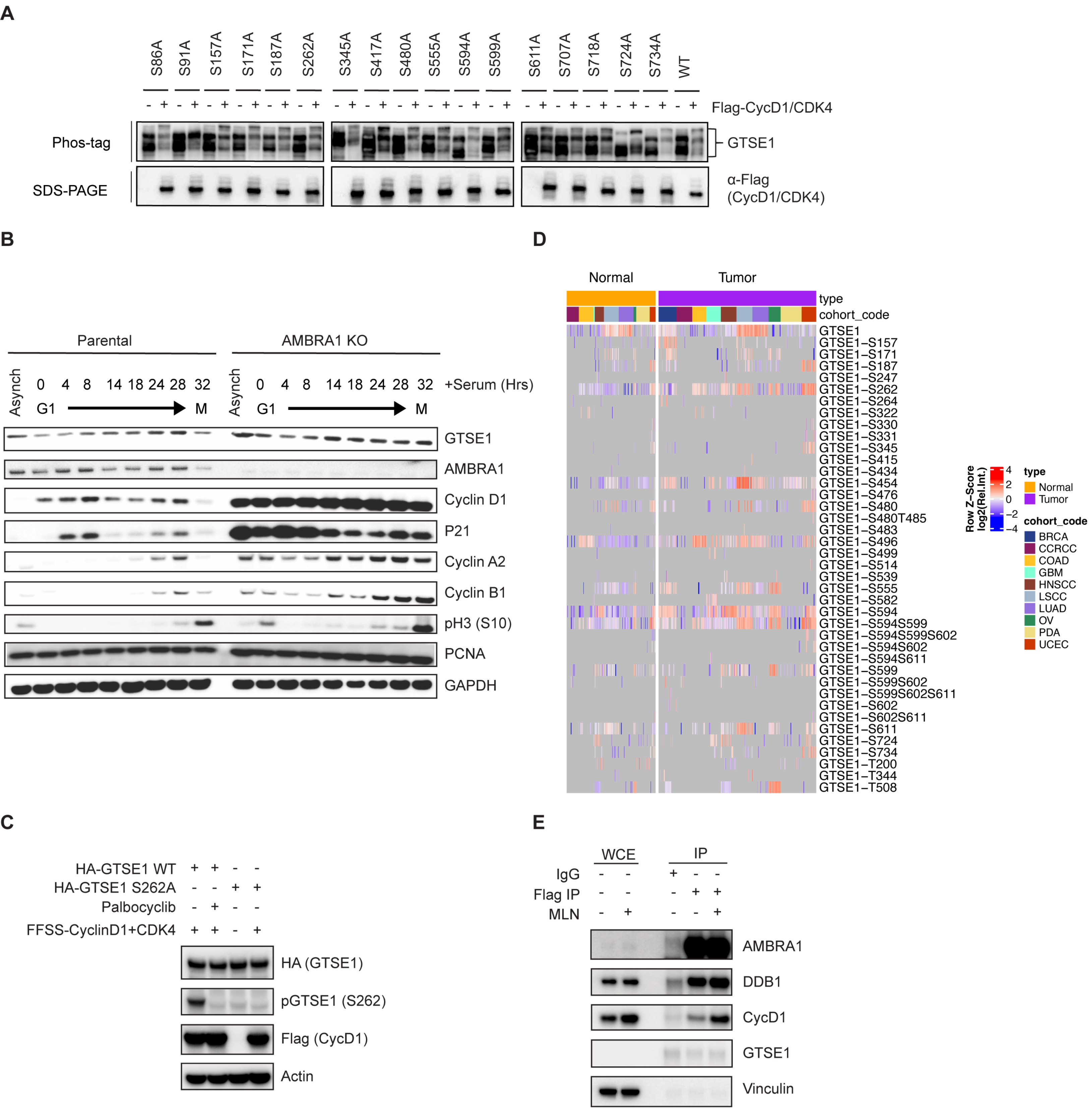

Figure Supplement 3 - García-Vázquez et al.

bioRxiv preprint doi: https://doi.org/10.1101/2024.06.26.600797; this version posted January 23, 2025. The copyright holder for this preprint (which was not certified by peer review) is the author/funder, who has granted bioRxiv a license to display the preprint in perpetuity. It is made available under aCC-BY 4.0 International license.

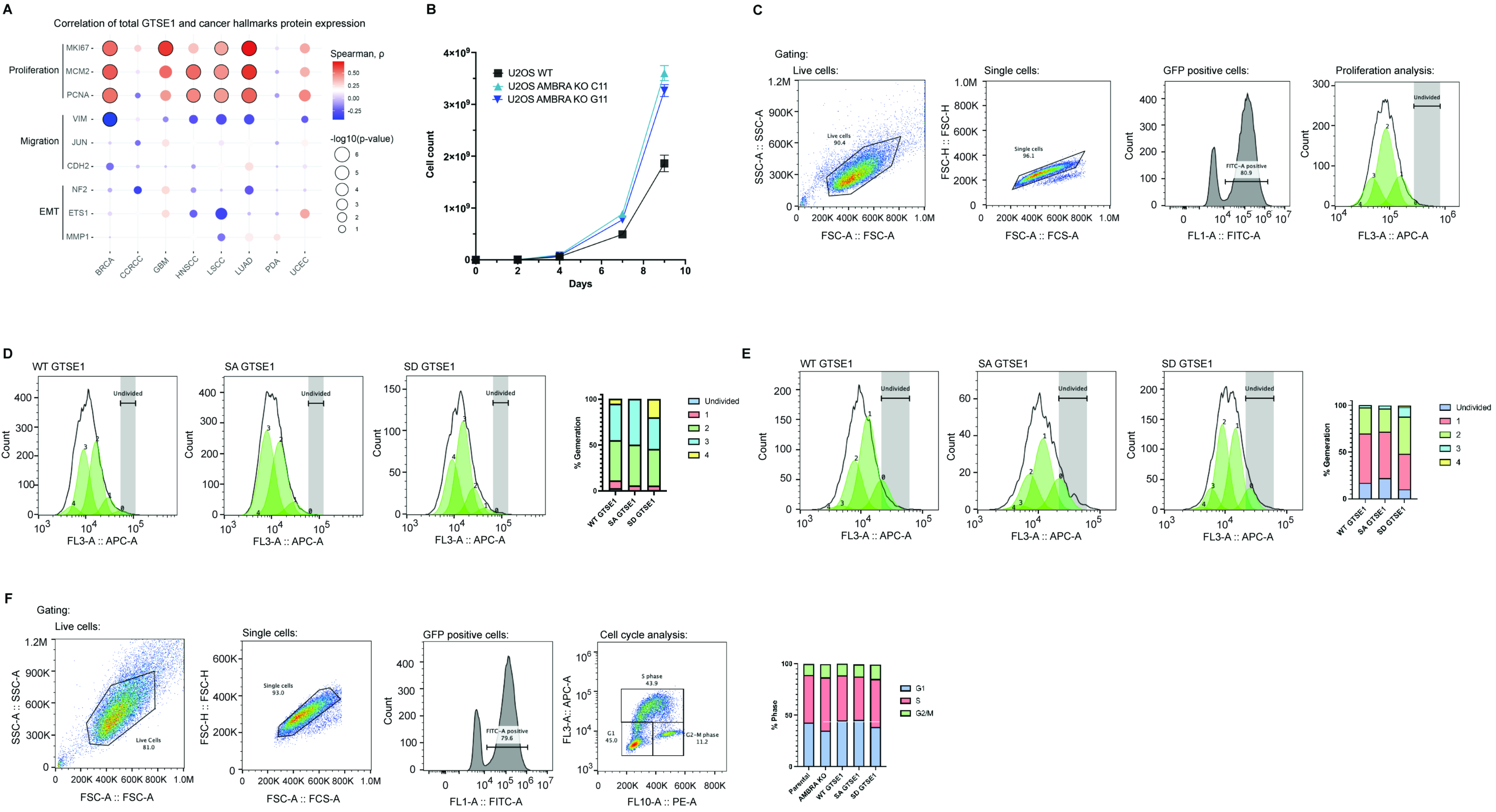

Figure Supplement 4 - García-Vázquez et al.
