# Supplementary material for: Stabilization of GTSE1 by cyclin D1-CDK4/6 promotes cell proliferation: relevance in cancer prognosis
Source: bioRxiv. 2024 Jun 27:2024.06.26.600797. Preprint. [Version 1] doi: 10.1101/2024.06.26.600797 (PMC11230433; doi:10.1101/2024.06.26.600797)
Supplement: Supplementary file 1 [file media-1.pdf]

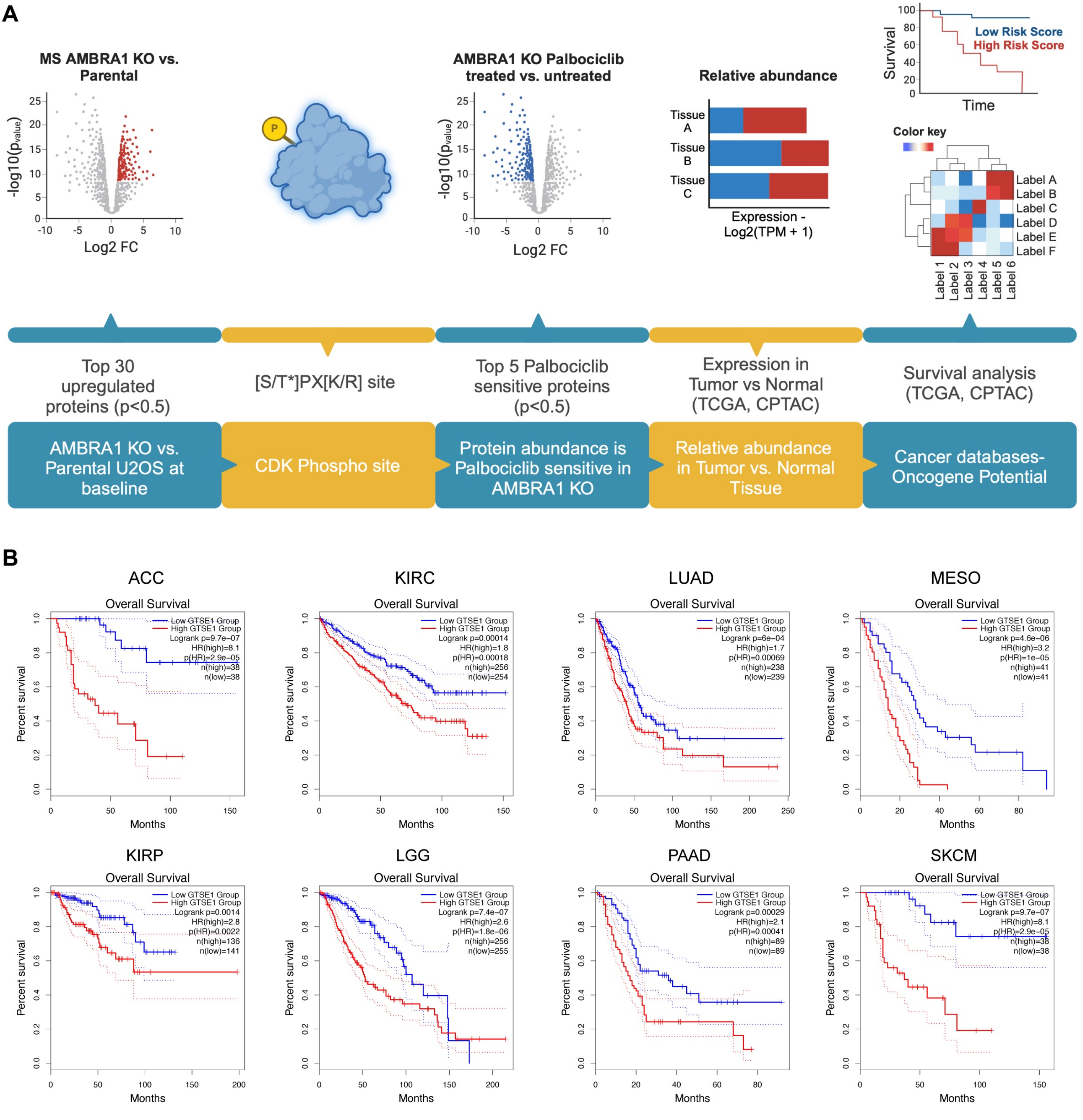


**Figure Supplement 1 - García-Vázquez et al.**

**A**

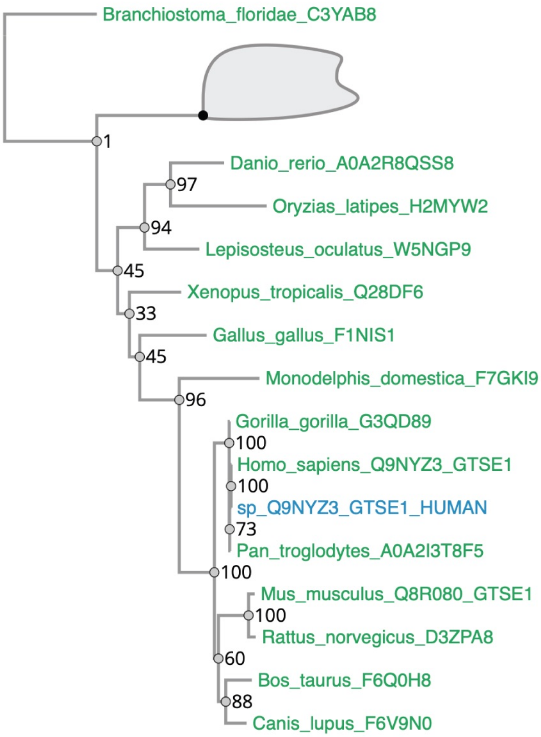


**B**

| Site | Species | Sequence |
|---|---|---|
| S86 | Homo sapiens | PPLPTSESPFAWSPL |
| | Rattus norvegicus | PLAPGSGSPCTWSPL |
| | Mus musculus | PLAPGSGSPCTLSPL |
| | Gallus gallus | KRVPISDEKLTWSPL |
| | Branchiostoma floridae | -----EEEKFVPLEL |
| S91 | Homo sapiens | SESPFAWSPLAGEKF |
| | Rattus norvegicus | SGSPCTWSPLTGEKF |
| | Mus musculus | SGSPCTLSPLTGEKF |
| | Gallus gallus | SDEKLTWSPLTGDKF |
| | Branchiostoma floridae | EEEKFVPLELNGDQY |
| S157 | Homo sapiens | KEKEMKKSPTSLKRE |
| | Rattus norvegicus | EQKRDKSPLSLKRE |
| | Mus musculus | KEQKRDRSPMSLKRE |
| | Gallus gallus | HH-IEKSPRAVKRE |
| | Branchiostoma floridae | KAPQNSPRNSPRRM |
| S171 | Homo sapiens | ETYYLSDSP--LLGPPV |
| | Rattus norvegicus | ETFCLPSS---RAQPLL |
| | Mus musculus | ETFCLPSS---RV-QPPM |
| | Gallus gallus | ETFCVGDSPacMLLPSFQ |
| | Branchiostoma floridae | MTYTLDT[5]ASssTTTADVQ |
| S187 | Homo sapiens | EPRLLASSPAL--PSSG |
| | Rattus norvegicus | EPQLLA-SPGLPNS- |
| | Mus musculus | EPQLLA-SPGLLSS- |
| | Gallus gallus | KESDKL----LPGDK |
| | Branchiostoma floridae | DSTQRQeNNEVKEGQ |
| S262 | Homo sapiens | PKKEIPASPSRTKIP |
| | Rattus norvegicus | LKKEVPASLQQTKPL |
| | Mus musculus | LKKEVPASIQRTKLV |
| | Gallus gallus | AKPGRQTNISTKRDS |
| | Branchiostoma floridae | IKPGI-------RK[16]A |
| S345 | Homo sapiens | GASSACTSPAVGKAK |
| | Rattus norvegicus | LVSGVGRSPTAGKAK |
| | Mus musculus | LESGVYRSSVAGKAK |
| | Gallus gallus | SVSSNLNSSL[4]IGKNG |
| | Branchiostoma floridae | TNPSQGSLGR[3]NGSID |
| S454 | Homo sapiens | RSIRRRDSCLNSKTK |
| | Rattus norvegicus | VSIKRQDSSLGCKTE |
| | Mus musculus | VSIKRRDSYLSCKTE |
| | Gallus gallus | GSTTKGSLCPKPRAR |
| | Branchiostoma floridae | AASANTSFSKKSLLP |
| S480 | Homo sapiens | PKFSIGDSPDSSTPK |
| | Rattus norvegicus | PQFSVGESPDRVTPK |
| | Mus musculus | PQFSVGESPGGVTPK |
| | Gallus gallus | PVKAGDITPNKSATK |
| | Branchiostoma floridae | PMRATLPMSQISTPK |
| S555 | Homo sapiens | TMPRAVGSPLCVPAR |
| | Rattus norvegicus | SMPRALVSPLCVPAR |
| | Mus musculus | SMPRALVSPLCVPAR |
| | Gallus gallus | TTPRTVFSPRIESVR |
| | Branchiostoma floridae | SSRRRSGIPTPRSMQ |
| S594 | Homo sapiens | DSRLVDVSPDRGSPP |
| | Rattus norvegicus | GQSQGLSSDESSSPP |
| | Mus musculus | GQAQGLSSDESSSPP |
| | Gallus gallus | ------SSEDDPSPP |
| | Branchiostoma floridae | SCLPTLAANDSPSEF |
| S599 | Homo sapiens | DVSPDRGSPPSRVPQ |
| | Rattus norvegicus | LSSDESSSPPSSVPQ |
| | Mus musculus | LSSDESSSPPSSVPQ |
| | Gallus gallus | *-SSEDDPSPPPVLPL |
| | Branchiostoma floridae | LAANDSPSEFPATHL |
| S611 | Homo sapiens | VPQALNFSPEESDST |
| | Rattus norvegicus | VPQALNFSPEKSDFP |
| | Mus musculus | VPQALNFSPEKSASP |
| | Gallus gallus | LPLMLDFSPEKAVI- |
| | Branchiostoma floridae | THLR----------- |
| S707 | Homo sapiens | NK-NVAKPSPV-VGQLI |
| | Rattus norvegicus | GRSDVGKPPKAELGQLI |
| | Mus musculus | GRNDVGKPAKAELGQLI |
| | Gallus gallus | NKVIPLKPVFSEQIKLI |
| | Branchiostoma floridae | ----------------- |
| S718 | Homo sapiens | GQLIDLSSPLIQLSP |
| | Rattus norvegicus | GQLIDLGSPLIQLSP |
| | Mus musculus | GQLIDLGSPLIQLSP |
| | Gallus gallus | IKLIDLSSPLITLSP |
| | Branchiostoma floridae | -------KPLIDFGE |
| S724 | Homo sapiens | SSPLIQLSPEADKEN |
| | Rattus norvegicus | GSPLIQLSPEADKEN |
| | Mus musculus | GSPLIQLSPEADKEN |
| | Gallus gallus | SSPLITLSPDINKEN |
| | Branchiostoma floridae | -KPLIDFGEEDMKTN |
| S734 | Homo sapiens | ADKENVDSPLLKF |
| | Rattus norvegicus | ADKENVDSPLLKF |
| | Mus musculus | ADKENVDSPLLKF |
| | Gallus gallus | INKENLDSPLLKF |
| | Branchiostoma floridae | DMKTN--SPAIAN |

**Figure Supplement 2 - García-Vázquez et al.**

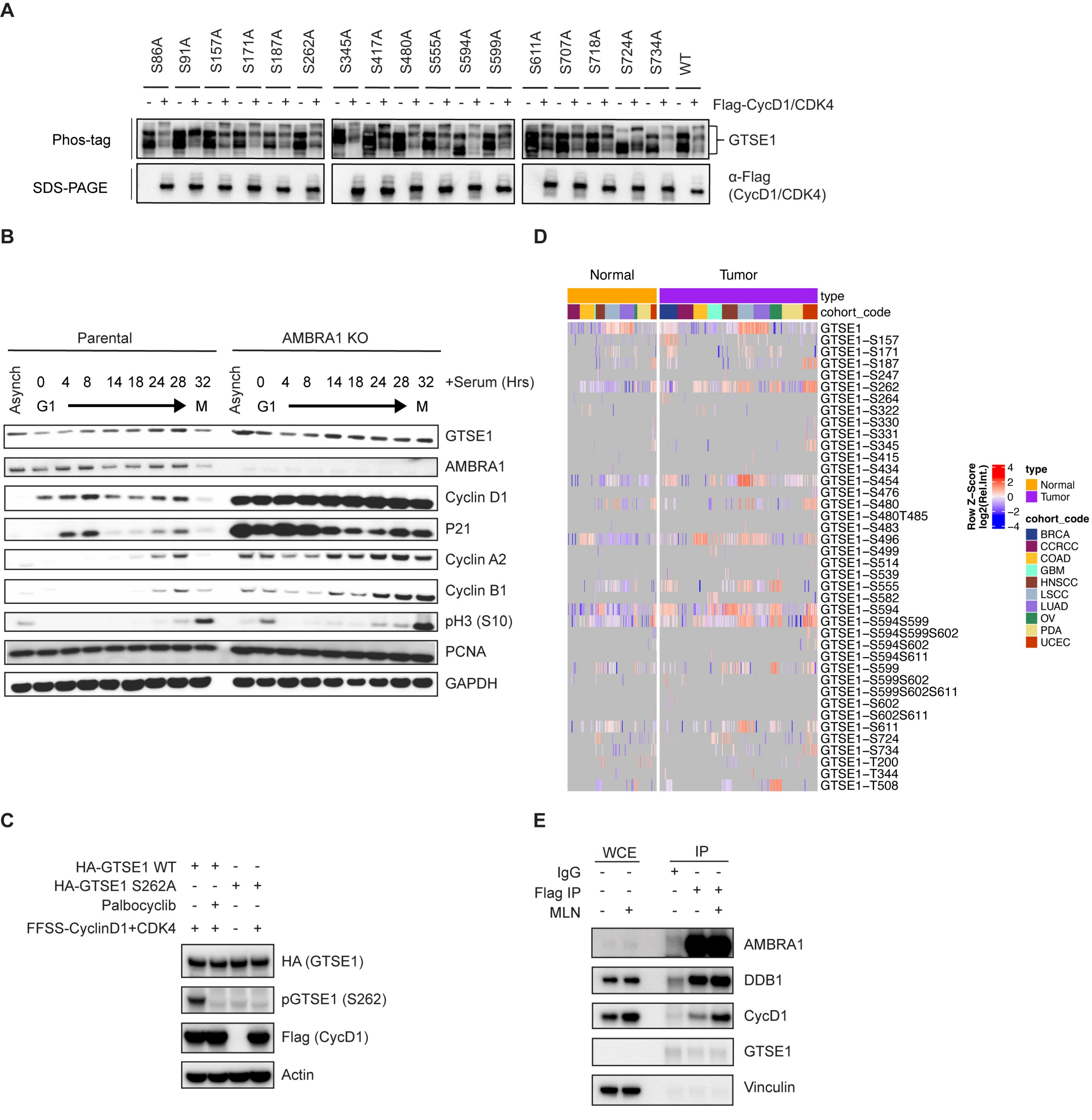


**Figure Supplement 3 - García-Vázquez et al.**

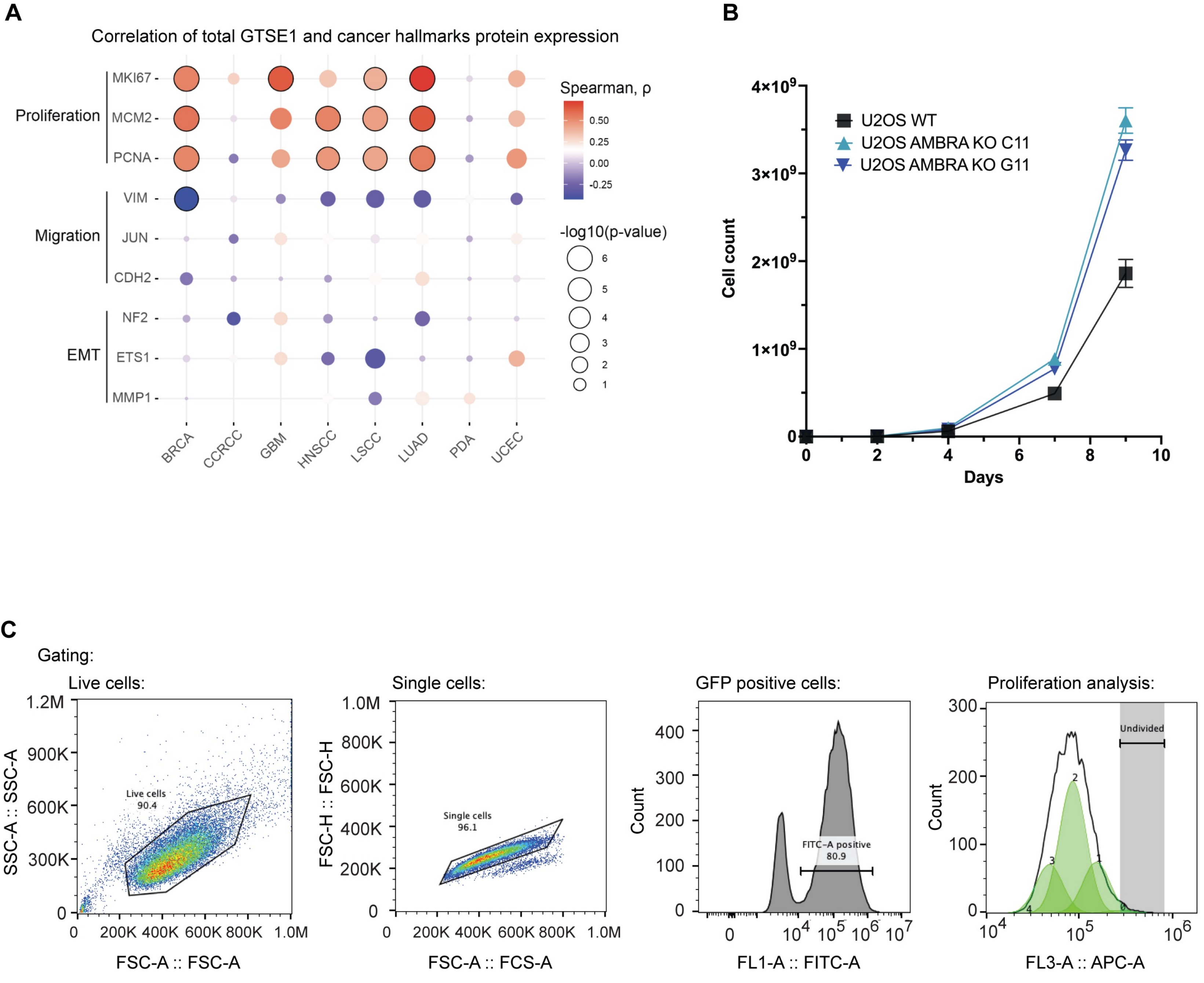


**Figure Supplement 4 - García-Vázquez et al.**
